# An Amyloid-Like Pathological Conformation of TDP-43 Is Stabilized by Hypercooperative Hydrogen Bonds

**DOI:** 10.3389/fnmol.2016.00125

**Published:** 2016-11-17

**Authors:** Miguel Mompeán, Marco Baralle, Emanuele Buratti, Douglas V. Laurents

**Affiliations:** ^1^Instituto de Química Física “Rocasolano”, Consejo Superior de Investigaciones Científicas (CSIC)Madrid, Spain; ^2^International Centre for Genetic Engineering and Biotechnology (ICGEB)Trieste, Italy

**Keywords:** pathological amyloids, transitive response DNA-bonding protein 43 kDa (TDP-43), hnRNPs, hyperstable H-bonds, amyotrophic lateral sclerosis (ALS), frontotemporal lobar degeneration, protein misfolding and disease

## Abstract

TDP-43 is an essential RNA-binding protein forming aggregates in almost all cases of sporadic amyotrophic lateral sclerosis (ALS) and many cases of frontotemporal lobar dementia (FTLD) and other neurodegenerative diseases. TDP-43 consists of a folded N-terminal domain with a singular structure, two RRM RNA-binding domains, and a long disordered C-terminal region which plays roles in functional RNA regulatory assemblies as well as pernicious aggregation. Evidence from pathological mutations and seeding experiments strongly suggest that TDP-43 aggregates are pathologically relevant through toxic gain-of-harmful-function and/or harmful loss-of-native-function mechanisms. Recent, but not early, microscopy studies and the ability of TDP-43 aggregates to resist harsh treatment and to seed new pathological aggregates *in vitro* and in cells strongly suggest that TDP-43 aggregates have a self-templating, amyloid-like structure. Based on the importance of the Gln/Asn-rich 341–367 residue segment for efficient aggregation of endogenous TDP-43 when presented as a 12X-repeat and extensive spectroscopic and computational experiments, we recently proposed that this segment adopts a beta-hairpin structure that assembles in a parallel with a beta-turn configuration to form an amyloid-like structure. Here, we propose that this conformer is stabilized by an especially strong class of hypercooperative hydrogen bonding unique to Gln and Asn sidechains. The clinical existence of this conformer is supported by very recent LC-MS/MS characterization of TDP-43 from *ex vivo* aggregates, which show that residues 341–367 were protected *in vivo* from Ser phosphorylation, Gln/Asn deamidation and Met oxidation. Its distinct pattern of SDS-PAGE bands allows us to link this conformer to the exceptionally stable seed of the Type A TDP-43 proteinopathy.

## Introduction

**Figure 1:**
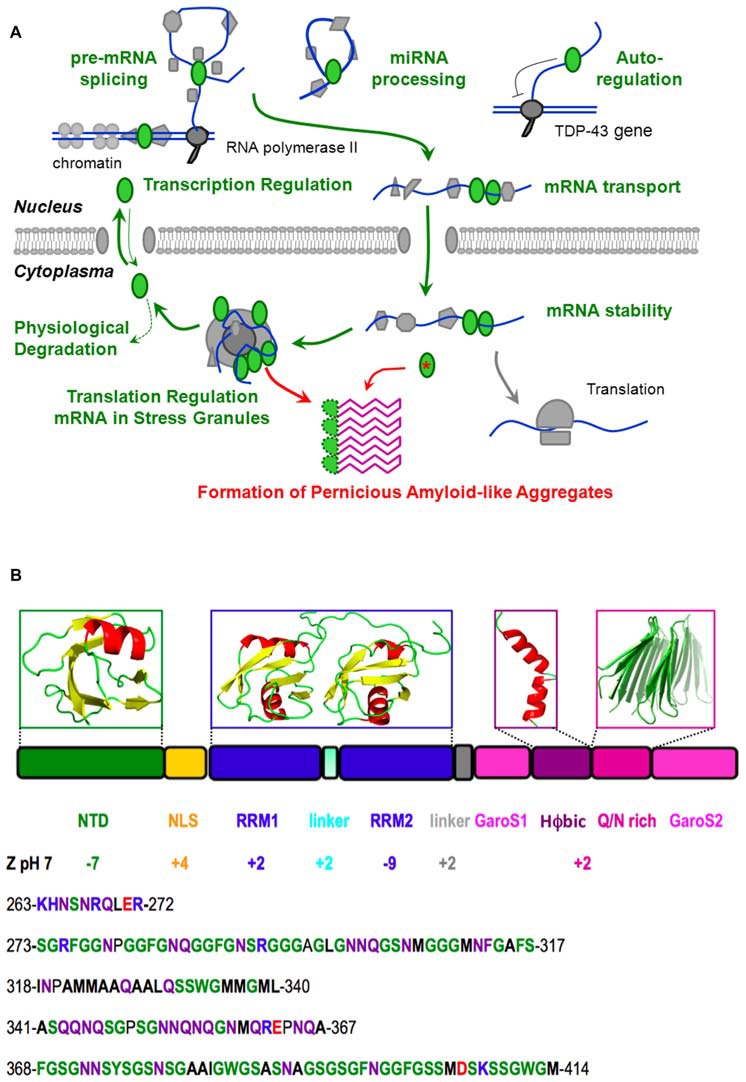
**Native function, pathological aggregation and structure of TDP-43. (A)** TDP-43 (*green ellipses*) performs vital physiological roles (*green*) in pre-mRNA splicing, microRNA processing, transcriptional regulation and post-transcriptional autoregulation of its own expression, mRNA transport, stability and translational regulation (through stress granule formation) before being degraded or shuttling back to the nucleus. However, the chance formation of aberrant conformers, which could be facilitated by mutation (*red asterisk*), aberrant cleavage, cytoplasmic mislocalization, aging and/or high local TDP-43 concentration in stress granules, may lead to the formation of a pathological amyloid-like aggregate. We propose that this aggregate consists of beta hairpins formed by residues 341–367 (*purple*) and is stabilized by hyper-stable inter-molecular hydrogen bonds. In this schematic diagram, DNA is represented by a *double blue line*, RNA is shown as a *curved blue line* and other proteins, the nuclear membrane and the ribosome are colored *gray*. **(B)** The domain composition and 3D structures of the N-terminal and RRM domains of TDP-43, as well as plausible, partially populated helical and quasi-amyloid conformers of the disordered C-terminal regions are shown. The net charge of the different domains, segments and the C-terminal region are indicated. The lower portion of the figure shows the sequence of the linker (residues 263–272), and the C-terminal subregions: G, S, aromatic-rich segment (GaroS1; 273–317), hydrophobic (318–340), Q/N-rich (341–367) and second GaroS region (368–414). Residues are colored* blue* (KHR), *red* (E, D), *green* (G, S, F, Y, W), *purple* (N, Q) or written in **bold** (aliphatics).

Amyotrophic lateral sclerosis (ALS) is a rare, paralyzing mortal disease which involves the death of motor neurons, leading to progressive muscle atrophy and death (Kiernan et al., 2011). In 1991, ubiquitinated aggregates were detected in the motor neurons of patients affected by ALS (Leigh et al., 1991). In 2006, these aggregates were identified as ubiquitinated and polyphosphorylated TDP-43 (Arai et al., 2006; Neumann et al., 2006), an essential, well-conserved protein involved in RNA splicing, transport and translation regulation (Buratti and Baralle, 2012). C-terminal fragments of 35 kD and 25 kD were also observed. Soon afterwards, many mutations in TDP-43 genetically linked to ALS and frontotemporal lobar dementia (FTLD) were discovered, as recently reviewed (Buratti, 2016). These mutations and the observation of TDP-43 aggregates in the motor neurons of practically all ALS and many FTLD patients are evidence of TDP-43’s central role in this spectrum of neuromuscular/dementia pathologies (Kiernan et al., 2011). From the point of view of the protein architecture, it is fascinating that almost all of these mutations are located in the C-terminal region (Pesiridis et al., 2009). In concert with mutational, functional and dysfunctional results from *in vitro* and *in vivo* studies, an advanced knowledge of TDP-43’s structure is key to understand its roles in physiological and pathological processes, which are summarized in Figure 1A, that could eventually guide the development of prophylactics and treatments.

### TDP-43 Contains Three Folded Domains and an Intrinsically Unfolded C-Terminal Region

Due to its intrinsic high-aggregation propensity, it has been impossible to solve the full structure of TDP-43. However, according to all the evidence gathered in recent years, TDP-43 consists of a folded N-terminal domain, two folded RRM domains and an intrinsically disordered C-terminal region (Figure 1B). The N-terminal domain has a singular tertiary structure, contains two negatively charged loops (Qin et al., 2014; Mompeán et al., 2016c) and is important for TDP-43’s physiological and pathological functions (Zhang et al., 2013; Budini et al., 2015; Sasaguri et al., 2016). In particular, the N-terminal domain is essential for the efficient capture of endogenous TDP-43 molecules into aggregates and induces a loss-of-function phenotype in cultured neuronal cells (Budini et al., 2015).

The RRM domains bind UG-rich segments of RNA and are essential for TDP-43’s physiological functions (Ayala et al., 2005; Lukavsky et al., 2013). Interestingly, both the N-terminal domain and the first RRM domain are stable (with T_M_’s close to 50°C; Kuo et al., 2009; Wang et al., 2013; Mompeán et al., 2016c), but the second RRM domain is much more stable (T_M_ = >85°C; Kuo et al., 2009; Wang et al., 2013). Although the physiological relevance of these differences are unknown; here we speculatively propose that the second RRM domain’s high stability could aid the proper presentation of the nuclear export signal present within this domain (residues 238–250) or chaperone residues 246–255 which tend to form amyloid and hydrogels (Wang et al., 2013; Saini and Chauhan, 2014). Among the similarities and differences between the two domains involved in nucleic acids, RRM2 adopts an atypical RRM-fold with an additional beta-strand that could mediate RRM2-RRM2 protein interactions.

The preferred binding motif of these RRMs is represented by single-stranded UG-rich regions/repeats that are recognized with high affinity (Buratti and Baralle, 2011; Polymenidou et al., 2011; Tollervey et al., 2011). Both RRMs contribute to nucleic acid binding, with RRM1 specifically recognizing one GU repeat. RRM2 recognizes one UG repeat and binds to additional nucleotides nonspecifically. Thus, the emerging binding consensus sequence is 5′-N(N)GUGN(N)UGN-3′. A unique feature of the TDP-43—RNA complex is the reversed binding of the UG-rich RNA with the 5′end being bound to RRM1, rather than RRM2 (Lukavsky et al., 2013). This is in contrast to all other tandem RRMs characterized to date and might explain some peculiarities of TDP-43 functions. In fact, whilst TDP-43 binding to UG-rich RNA through RRM1 shows a very clear correlation between binding and inhibitory splicing function, this does not occur for RRM2 where alanine mutations in its recognition sites have only a small effect on TDP-43’s overall RNA binding affinity. Nevertheless, these interactions are functionally important and are required for the splicing function of TDP-43. Thus, the role of RRM2 could be to orient the UG-rich RNA for productive TDP-43 dimerization on pre-mRNA target sites.

Another remarkable feature of the second RRM domain is that it carries an overall charge of −9 at pH 7. In contrast, the first RRM domain has a net positive charge (Figure 1B). This difference might account in part for the second RRM domain’s weaker RNA binding as its negative charges could electrostatically repel longer RNA oligos. In this respect, it is interesting to note that the second RRM domain binds shorter RNA oligos better than longer ones (Kuo et al., 2009).

In contrast to the N-terminal and RRM domains, the C-terminal region is intrinsically disordered (Lim et al., 2016). It is remarkably poor in charged residues (Figure 1B), which is related to its meager solubility (Mompeán et al., 2016a). It can be divided into four sub-regions; namely a Gly, Aromatic, Ser-rich (GaroS) segment, a hydrophobic segment, a Gln/Asn (Q/N)-rich segment, and a second GaroS rich segment. The GaroS segments resemble the [G,S]Y[G,S] motifs of the intrinsically disordered region of “Fused in Sarcoma” (FUS), an RNA-binding protein that like TDP-43 is also aberrantly aggregated in ALS and FTLD pathologies. These motifs were proposed to endow TDP-43 and FUS with the ability to participate in RNA granules and form hydrogels (Kato et al., 2012). Although these segments do not resemble those of pathological amyloids, their ability to increase the local concentration of TDP-43 within RNA granules may boost the probability that the aberrant oligomers might form, along the lines of the model recently proposed for FUS (Patel et al., 2015), particularly in the context of some disease-related mutations (Lim et al., 2016).

These considerations raise several hypotheses for the origin of the pathology that involve a crucial role for interdomain contacts to eventually trigger the pathological aggregation process. In this respect, Zhang et al. (2013) have modeled the possible interaction between the N-terminal and C-terminal domains to regulate proper homodimerization of TDP-43 while Mackness et al. (2014) have investigated the different potential folding intermediates in the RRM1 and RRM2 domains. In addition, Wang et al. (2013) have shown that TDP-43 without the C-terminal region can form a homodimer primarily via N-terminal domain contacts.

Although the previously mentioned genetic evidence and these biochemical data strongly link TDP-43’s C-terminal region to pathology, many aspects of this issue still remain to be addressed. For example, there is still a very open debate regarding the amyloid vs. amorphous nature of these aggregates, their atomic level structure, and what interactions could stabilize them. In this Perspective, we shall put together diverse results from the literature to address these points and advance a working hypothesis that an amyloid-like conformer, composed of beta-hairpins formed by residues 341–357, is the relevant pernicious structure for TDP-43 Type A proteinopathies.

### TDP-43 can Form Amyloid-Like Fibrils which Seem to be Pathologically Relevant

One important and long-standing issue is whether the TDP-43 aggregates seen in diseased tissues are amyloid-like or not.

First, it has to be acknowledged that early studies generally found that TDP-43 in cell inclusions from *ex vivo* tissues did not bind amyloid specific dyes like Thioflavin S or show much fibril formation when examined by immunostaining and optical microscopy (Cairns et al., 2007; Neumann et al., 2007). Furthermore, when TDP-43 is expressed in bacteria and purified from inclusion bodies (Capitini et al., 2014) or expressed in yeast (Johnson et al., 2008) no evidence for amyloid formation was detected. Moreover, TDP-43 produced from human cells or *E. coli* did not bind Thioflavin T or Congo Red, although it did form highly cytotoxic oligomers (Fang et al., 2014).

In contrast, several studies found that short peptides derived from TDP-43’s second RRM domain and C-terminal region adopt fibril structures that bind amyloid-specific dyes (Chen et al., 2010; Guo et al., 2011; Saini and Chauhan, 2011, 2014; Jiang et al., 2013; Mompeán et al., 2014; Sun et al., 2014; Zhu et al., 2014).

Finally, even middle-of-the-road positions have been described. For example, Wang et al. (2013) found that C-terminal truncated forms of TDP-43 can adopt well defined fibril structure which nonetheless does not bind amyloid specific dyes or antibodies.

Of course, in most cases these studies’ relevance for human pathology still needs to be demonstrated. It has to be taken in consideration, in fact, that TDP-43 is not naturally present in lower organisms and therefore its conformation could be altered by diverse *in vivo* environments. Likewise, the different context of isolated polypeptide segments vs. their presence within the complete protein has to be considered. Nonetheless, studies of *ex vivo* patient tissues using higher resolution immuno-gold labeling and electron microscopy did uncover solid evidence for amyloid-like fibrils formed by TDP-43 in ALS/FTLD affected neurons (Thorpe et al., 2008) as well as cells affected by other neurodegenerative diseases (Lin and Dickson, 2008). More recently, an optical microscopy study using Thioflavin S and optical microscopy has reported evidence for amyloid formation by TDP-43 in 30% of ALS patients (Robinson et al., 2013). Finally, upon treating tissues to remove background due to lipid autofluorescence, TDP-43 amyloid-like structures could be detected by Thioflavin S fluorescence in the relevant tissues of most patients with ALS or FTLD (Bigio et al., 2013). In conclusion, these results are good evidence that TDP-43 in patients can form amyloid-like fibrils that are pathologically relevant.

### Hyperphosphorylation of TDP-43

One key hallmark of TDP-43 aggregates is hyperphosphorylation (Arai et al., 2006; Buratti and Baralle, 2012), and antibodies raised against specific phosphoserines enabled the identification of phosphorylated S409 and S410 as characteristic signatures of pathological TDP-43 aggregates (Neumann et al., 2009). These negatively charged phosphates seem to decrease the protein’s aggregation (Brady et al., 2011; Li et al., 2011) and phosphorylation appears to occur after aggregation *in vivo* as the cell attempts to solubilize and degrade the inclusions.

Additional phosphorylation sites were identified utilizing a set of specific antibodies (Hasegawa et al., 2008) and mass spectrometry (Kametani et al., 2009). Most phosphorylated residues (18 of 29) are in the C-terminal region, including S342, S347 and S350 in the Q/N-rich subregion. More recently, this laboratory has also analyzed TDP-43 aggregates from two *ex vivo* ALS brains (Kametani et al., 2016). Remarkably, they found 13 phosphorylation sites in one brain and 15 sites in the second. By comparing these results to those obtained using soluble TDP-43 (Kametani et al., 2009), it is clear that some serines; namely S342, S347, and S350, seem to be hidden from kinase and thus protected from phosphorylation in the context of the pathological inclusions. This observation is particularly important as it may give a clue about the location of the aggregation core.

### The Formation of a Helix in the Hydrophobic C-Terminal Segment Could Modulate Toxic Aggregate Formation

The relevance of the hydrophobic and Q/N-rich segments to TDP-43 aggregation was demonstrated by Jiang et al. (2013) who observed that the *C. elegans* homolog of TDP-43, which lacks these segments, is much less prone to aggregation.

The hydrophobic stretch has a very weak tendency to form helix in aqueous solution, as evidenced by very small ^1^Hα, ^13^Cα conformational chemical shifts and a lack of ^1^HN_i_-^1^HN_i+1_,_i+3_,_i+4_ NOES (Mompeán et al., 2015); nonetheless, a significant amount of helix does form in the presence of TFE or DPC micelles (Jiang et al., 2013; Lim et al., 2016). These are important results as pathological amyloid formation is often characterized by a loss of native helix in favor of a gain in beta structure. In agreement with these findings, evidence for a helix to beta conformational conversion was found in this hydrophobic segment (Jiang et al., 2013).

Our own recent results (Budini et al., 2012; Mompeán et al., 2014, 2015) also point to the Q/N-rich segment as being very important for TDP-43 aggregation and amyloid formation. Moreover, the recent mass-spectrometry/proteolysis study of TDP-43 from *ex vivo* brains of two ALS patients provides insight into the relative importance of the hydrophobic and Q/N rich regions (Kametani et al., 2016). In this report, Kametani and co-workers found that residues 341–346 or 341–360 of the Q/N-rich segment were protected against proteolytic cleavage, deamidation and phosphorylation (in patient 1 and patient 2, respectively). Although more cases are needed to confirm whether this is indeed a general pathological feature, these data are consistent with these segments forming an highly stable amyloid-like structure, such as the one we have proposed (Mompeán et al., 2015), which protects them against cleavage and modification. In contrast, residues of the hydrophobic segment are heavily modified by Ser phosphorylation, Gln deamidation and Met oxidation, which suggests they are exposed in patient aggregates. This implies that the hydrophobic segment does not form an amyloid structure which would prevent these modifications.

### The Q/N-Rich Segment of TDP-43’s C-Terminal Region can Quickly form a Stable, Amyloid-Like Structure

Regarding TDP-43 aggregation, the Q/N-rich segment is especially interesting because variants lacking it or substituting a polyAla segment do not aggregate (Budini et al., 2012). We have recently investigated this segment’s conformational transitions using CD/NMR spectroscopies and computational methods. Our studies have shown that the Q/N-rich segment is initially unfolded but over the course of several hours it converts into a structure rich in beta structure which binds thioflavin T (Mompeán et al., 2014). This segment, modeled as a beta hairpin, is stable in long molecular dynamics simulations. Additional studies, using conformational antibodies specific for amyloid, solid state NMR, X-ray diffraction and electron microscopic, confirmed the ability of the Q/N-rich segment of TDP-43 to adopt an amyloid-like structure, whose fibrils are broad and tend to associate laterally, like those of polyglutamine (Mompeán et al., 2015).

**Figure 2:**
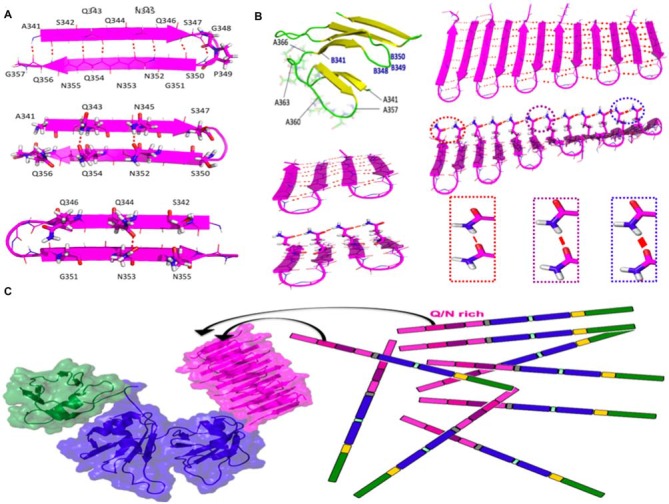
**Structure and stabilizing H-bonds in the amyloid-like conformer of TDP-43 composed of residues 341–366. (A)** Schematic diagram of the beta-hairpin adopted by residues 341–357 of TDP-43. **(B)** Residues 358–366 could act as a loop to link different beta-hairpins in the same beta-sheet or between different beta sheets (*yellow strands and green loops*). One of the two sheets of the 4X Q/N (4 hairpins) or of 12X Q/N (6 hairpins) are shown in *magneta*. Cooperative and hyperpolarization effects greatly fortify Q/N side chain H-bonds (*red lines*) and moderately strengthen backbone H-bonds (*dashed red lines*) in longer 12X Q/N assembles (*right*) but not the shorter 4X Q/N sheet (*left*). The thickness of the red lines reflects the strength of the H-bonds. **(C)** The formation of exceptionally stable H-bonds provides a free energy sink which drives the binding of endogenous TDP-43 molecules to the quasi-amyloid structure formed by the 12X Q/N TDP-43 construct. The color code of the domains matches that of the sequence diagram in Figure 1 and the structures were modeled according to Mompeán et al. (2014, 2015) for the Q/N segments (magenta), Mompeán et al. (2016c) for the NTD domain (green) and Lukavsky et al. (2013) for the RRM domains (blue).

Using computational tools, we built up an amyloid-like structural model based on beta-hairpins composed of residues 341–357 of TDP-43 arranged in parallel and with a beta-turn topology. This arrangement was found to be stable in long MD simulations (Figure 2). By contrast, other configurations with consecutive beta hairpins arranged in an anti-parallel fashion or with beta-arc topology were unstable (*not shown*). Based on this model, the efficient aggregation of the 12X Q/N TDP-43 construct *in vivo* suggests that these 12 repetitions are likely to assemble into two stacked beta sheets, each containing six beta-strands or 12 beta strands that form the nascent, quasi-amyloid core. Residues 358–366 act as a linker to connect 341–357 beta-hairpins and allow proper cross-beta spine formation. This segment could connect the beta-hairpins within each sheet, and link the two sheets, allowing them to pack against each other (Figure 2B; Mompeán et al., 2015).

Based on the apparent protection of residues 341–367 against Ser phosphorylation, Gln/Asn deamidation and Met oxidation in TDP-43 *ex vivo* aggregates, we therefore propose as a working hypothesis that our conformational model for this segment (Figure 2) may correspond to the pathological conformer present in the *ex vivo* aggregates of patient 2 described by Kametani et al. (2016). Indeed, based on the similarities of the characteristic bands in SDS-PAGE chromatograms previously described (Nonaka et al., 2013; Kametani et al., 2016), our conformer may represent the structural core of Type A TDP-43 proteinopathy, the most common class of TDP-43 maladies (Mackenzie et al., 2011). As TDP-43 Type A aggregates are remarkably more heat-stable as seeds than Type B or Type C aggregates (Nonaka et al., 2013), what remains to be addressed is the basis of this exceptional stability. Below, we present a model that can account for this.

### Hyperpolarization-Mediated Stabilization of Gln and Asn Side Chain Hydrogen Bonds can Account for the Remarkable Stability of the Putative Amyloid Formed by the Q/N-Rich Segment of TDP-43

We have recently compared hydrogen bond cooperativity in a Q/N-rich amyloid, the paradigmatical GNNQQNY heptapeptide form the yeast prion Sup35, to the hydrophobic amyloid M_35_VGGVV_40_ from Aβ (Mompeán et al., 2016b). Whereas the backbone amide groups of both Q/N-rich and hydrophobic amyloids experience stabilization due to hyperpolarization, an additional and stronger type of hyperpolarization occurs in Asn and Gln side chain amides (Figure 2B). The strengthening due to hyperpolarization increases with the number of strands.

According to this working hypothesis, hydrogen bond hyperpolarization provides a very favorable free energy change for binding monomers to the quasi-amyloid structure formed by the Q/N-rich segment, thus permitting the TDP-43-12X Q/N construct to efficiently sponge up endogenous TDP-43 (Figure 2C). Hydrogen bond hypercooperativity can also explain how the Q/N-rich subregion enables TDP-43 to bind and co-aggregate with polyglutamine (Fuentealba et al., 2010). As a side note, this also provides a logical explanation for why TDP-43 12X Q/N repeats readily form a stable amyloid capable of sequestering endogenous TDP-43 whereas a stoichiometrically equivalent amount of Q/N repeats in the context of 2X Q/N or 4X Q/N constructs does not (Budini et al., 2012).

Recruitment of endogenous TDP-43 into aggregates within this cellular model can be tentatively attributed to two different processes. First, the tandem repetitions could form an amyloid fold that might sponge up and sequester nuclear TDP-43 through its Q/N segment. Second, heterodimerization via the NTDs of endogenous TDP-43 with TDP-43-12X Q/N would increase the local concentration of the Q/N rich segments and thereby favor their self-association (Romano et al., 2015). In the future, more complete structural models of TDP-43 could help determine whether these processes act antagonistically, independently or synergistically.

Recent studies strongly suggest that, once formed, pernicious conformers of TDP-43 could spread through the nervous system in a prion-like fashion (Nonaka et al., 2013; Smethurst et al., 2015). This issue will likely prove crucial for a better understanding of TDP-43 proteinopathies. In fact, the aggregates’ size, location, toxicity and their stability have been advanced as factors accounting for the efficiency of prion-like spreading by pernicious protein aggregates (Espargaró et al., 2016). In these conditions, the exceptional stability of our conformer/Type A could well be pertinent to provide a first structural framework for future studies aimed at further elucidating this issue.

## Author Contributions

MM, MB, EB and DVL conceived the ideas, wrote the manuscript, critically revised it and approved the final version for publication. All four authors agree to be accountable for the contents.

## Conflict of Interest Statement

The authors declare that the research was conducted in the absence of any commercial or financial relationships that could be construed as a potential conflict of interest.
